# Supplementary material for: Isolation of Hepatic and Adipose-Tissue-Derived Extracellular Vesicles Using Density Gradient Separation and Size Exclusion Chromatography
Source: Int J Mol Sci. 2023 Aug 11;24(16):12704. doi: 10.3390/ijms241612704 (PMC10454538; doi:10.3390/ijms241612704)
Supplement: Supplementary file 1 [file ijms-24-12704-s001.zip › ijms-2410757-supplementary.pdf]

Supplementary Materials

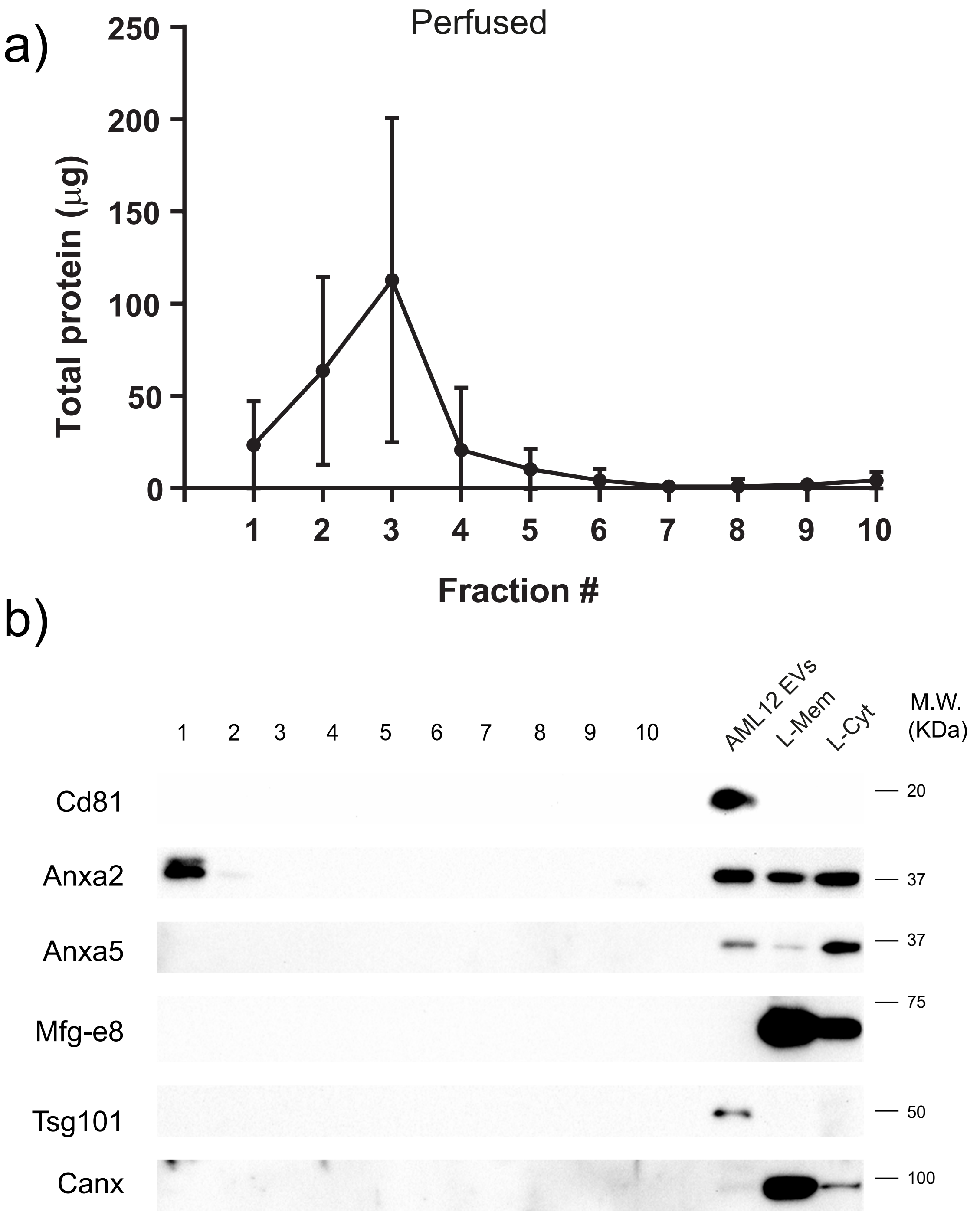

Figure S1. Isolation of hepatic-derived extraellular vesicles from perfused tissue. To evaluate the contribution of blood components in the EV preparations, mice were transcardially perfused with 0.9% NaCl before organ dissection. (**a**) Total protein per fraction obtained after ultracentrifugation of perfused hepatic tissue digested with papain. Perfusion reduced the amount of recovered protein after density gradient ultracentrifugation . (**b**) Western blot analysis for EV markers of density gradient fractions from perfused tissue. We only detected Anxa2 in two fractions while the rest of the markers were out of our detection range. As positive control we used EVs isolated form the hepatic cell line AML12 and liver homogenates enriched for membrane proteins (L-Mem) and cytosolic proteins (L-Cyt).

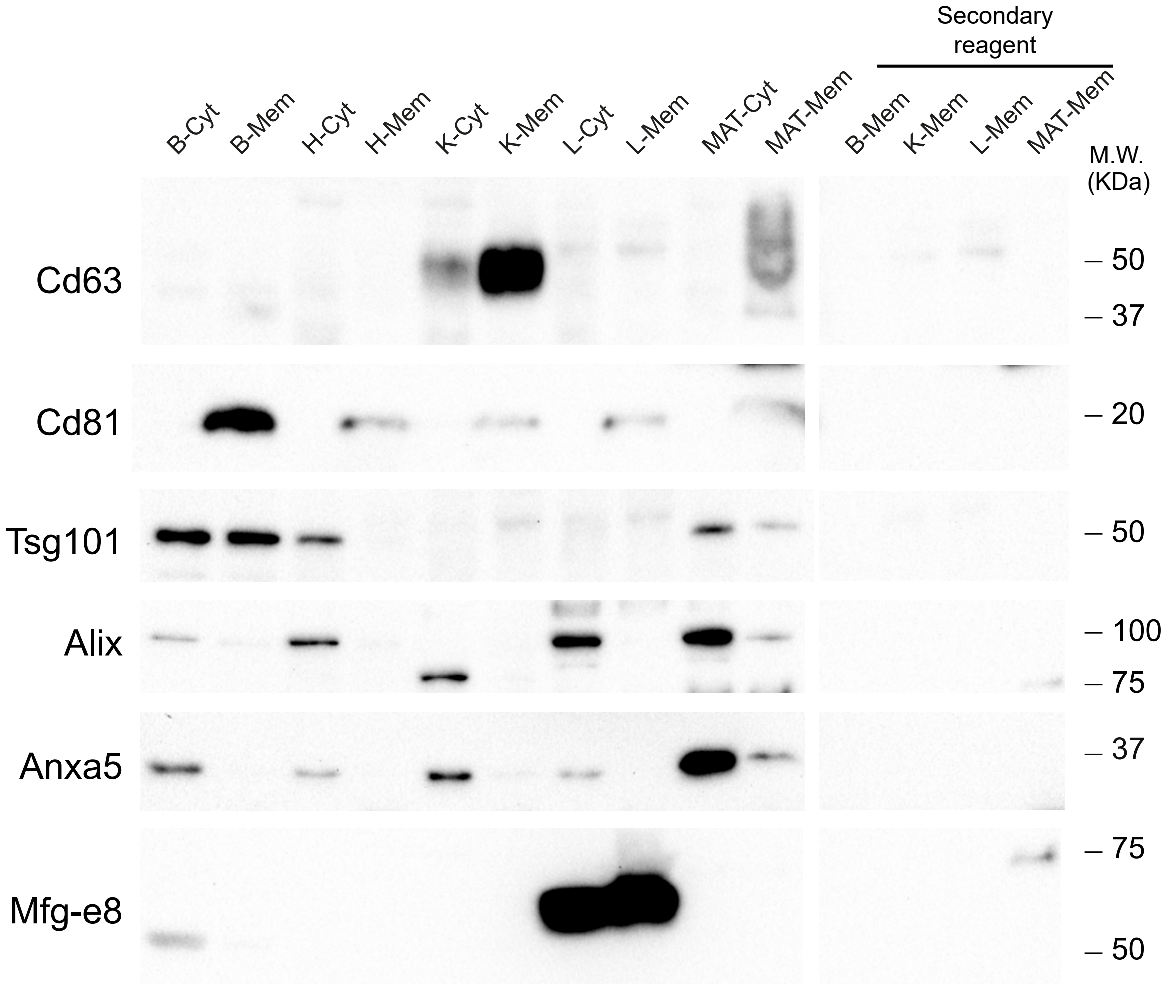

Figure S2. Differential levels of EV markers in brain, heart, kidney, liver, and mesenteric adipose tissue. Membrane and cytosolic proteins were enriched using the MEM-PER Plus Membrane Protein Extraction Kit from 0.5g of each tissue. All lanes were loaded with 50 μg of protein with exception of mesenteric adipose tissue (MAT) lanes where we loaded 25 μg in membrane (MAT-Mem) and cytosolic (MAT-Cyt) protein enriched fraction. Note that the liver did not contain detectable levels of Cd63, showed low levels of Tsg101, and displayed the highest levels of Mfg-e8. MAT-Mem presented high levels of Cd63. For all the membranes homogenates we tested for background due to secondary reagent employed for each primary antibody: IgG κ-binding protein for Cd81, Tsg101, Alix, Anxa5, and Mfg; and anti-rabbit for Cd63. B-Cyt = brain cytosolic proteins, B-Mem = brain membrane proteins, H-Cyt = heart cytosolic proteins, H-Mem = heart membrane proteins, L-Cyt = liver cytosolic proteins, L-Mem = liver membrane proteins.
